# Increase in participation but decrease in performance in age group mountain marathoners in the ‘Jungfrau Marathon’: a Swiss phenomenon?

**DOI:** 10.1186/s40064-015-1330-y

**Published:** 2015-09-18

**Authors:** Beat Knechtle, Thomas Rosemann, Matthias A. Zingg, Christoph A. Rüst

**Affiliations:** Gesundheitszentrum St. Gallen, Vadianstrasse 26, 9001 St. Gallen, Switzerland; Institute of Primary Care, University of Zurich, Zurich, Switzerland

**Keywords:** Female, Male, Runner, Master

## Abstract

Participation and performance trends for age group marathoners have been investigated for large city marathons such as the ‘New York City Marathon’ but not for mountain marathons. This study investigated participation and trends in performance and sex difference in the mountain marathon ‘Jungfrau Marathon’ held in Switzerland from 2000 to 2014 using single and mixed effects regression analyses. Results were compared to a city marathon (Lausanne Marathon) also held in Switzerland during the same period. Sex difference was calculated using the equation ([race time in women] − [race time in men]/[race time in men] × 100). Changes in sex differences across calendar years and were investigated using linear regression models. In ‘Jungfrau Marathon’, participation in all female and male age groups increased with exception of women in age groups 18–24 and men in age groups 30–34, 40–44 and 60–64 years where participation remained unchanged. In ‘Lausanne Marathon’, participation increased in women in age groups 30–34 to 40–44 years. In men, participation increased in age groups 25–29 to 44–44 years and 50–54 years. In ‘Jungfrau Marathon’ runners became slower across years in age groups 18–24 to 70–74 years. In ‘Lausanne Marathon’, runners became slower across years in age groups 18–24 and 30–34 to 65–69 years, but not for 25–29, 70–74 and 75–79 years. In ‘Jungfrau Marathon’, sex difference increased in age groups 25–29 (from 4 to 10 %) and 60–64 years (from 3 to 8 %) but decreased in age group 40–44 years (from 12 to 6 %). In ‘Lausanne Marathon’, the sex difference showed no changes. In summary, participation increased in most female and male age groups but performance decreased in most age groups for both the mountain marathon ‘Jungfrau Marathon’ and the city marathon ‘Lausanne Marathon’. The sex differences were lower in the ‘Jungfrau Marathon’ (~6–7 %) compared to the ‘Lausanne Marathon’ where the sex difference was ~10–12 % from age groups 18–24 to 55–59 years. These unexpected findings might be a typical Swiss phenomenon. Future studies need to investigate whether this trend can also be found in other endurance sports events held in Switzerland and other mountain marathons held in other countries.

## Background

Marathon running is of high popularity where marathon races are mainly held as city marathons in large cities (Jokl et al. 2004; Lepers and Cattagni 2012; Leyk et al. 2007, 2009, 2010). Participation and performance trends in elite and age group city marathoners are well investigated (Ahmadyar et al. 2015; Anthony et al. 2014; Aschmann et al. 2013; Cribari et al. 2013, Jokl et al. 2004, Lepers and Cattagni 2012; Leyk et al. 2007, 2009), but very little is known for mountain marathoners (Zingg et al. 2013).

In a large city marathon such as the ‘New York City Marathon’, most of the successful finishers are age group or master runners (Jokl et al. 2004), defined as athletes older than 35 years (Reaburn and Dascombe 2008). In the ‘New York City Marathon’, participation increased in master age groups between 1983 and 1999 at a higher rate compared to other age groups and master runners improved their race times at a greater rate compared to younger athletes (Jokl et al. 2004). Lepers and Cattagni (2012) investigated for the ‘New York City Marathon’ participation and performance trends in master runners during a longer period from 1980 to 2009. During this period, the participation of male master runners increased to a greater extent compared to female master runners. Male master runners older than 64 years and female master runners older than 44 years improved their performance (Lepers and Cattagni 2012).

Apart from marathon running in a city marathon, athletes can also compete in mountain marathons (http://www.mountainrunning.com, http://www.wmra.info). However, in contrast to city marathons (Jokl et al. 2004, Lepers and Cattagni 2012, Leyk et al. 2007, 2009, 2010) we have no data on the participation and performance trends in age group mountain marathoners. Since mountain running is of increasing popularity (http://www.mountainrunning.com, http://www.wmra.info), the aim of this study was to investigate participation and performance trends in age group marathoners competing in the ‘Jungfrau Marathon’ held in the Swiss Alps. This marathon race started in 1993 and is actually the most famous mountain marathon in Europe. In 2007 and 2012, the ‘Jungfrau Marathon’ was held as the World Championship in mountain running. Based upon the findings for a large city marathon such as the ‘New York City Marathon’, we hypothesized that age group mountain marathoners competing in the ‘Jungfrau Marathon’ would increase their participation and improve their performance. To compare findings for this mountain marathon, we analyzed also race data from a city marathon (Lausanne Marathon) held during the same time period and in the same country.

## Methods

### Ethics

All procedures used in the study were approved by the Institutional Review Board of Kanton St. Gallen, Switzerland with a waiver of the requirement for informed consent of the participants given the fact that the study involved the analysis of publicly available data.

### The races

In this study, all athletes who finished the ‘Jungfrau Marathon’ and the ‘Lausanne Marathon’ between 2000 and 2014 were analysed for participation, running time, age, and sex.The data set for this study was obtained from the race website http://www.jungfrau-marathon.ch for the ‘Jungfrau Marathon’ and from http://de.lausanne-marathon.com for the ‘Lausanne Marathon’.

The ‘Jungfrau Marathon’ in Switzerland started in 1993, after mountain running was becoming increasingly popular in Europe. Nowadays, the ‘Jungfrau Marathon’ is one of the most popular mountain marathons in the world. The race is held annually in autumn. The ‘Jungfrau Marathon’ starts in Interlaken (565 m above sea level) and finishes at the ‘Kleine Scheidegg’ (2095 m above sea level). The course covers 1830 m of altitude gain and 305 m of loss in altitude. The first quarter of the race is mostly flat and only ~300 m of altitude difference are covered up to the first half of the marathon. The end of the race stands at 2095 m above sea level, right next to the world famous Eiger. In 2007 and 2012, the ‘Jungfrau Marathon’ was the official World Championship in mountain running, which is held annually along a different race each year.

The ‘Lausanne Marathon’ started in 1992 and is the second most important city marathon in Switzerland behind the ‘Zürich Marathon’. The race is held annually in autumn in the city of Lausanne, on the border of Lake Léman, on a flat course. After the first half, the course turns and goes back to Lausanne. Overall, the race has minus 36 meters of altitude change. Apart from the marathon, the ‘Lausanne Marathon’ hosts the annual semi-marathon Swiss Championship, a quarter marathon race, and a mini race for kids. All these races make the ‘Lausanne Marathon’ one of the most important marathon events held in Switzerland.

### Statistical analysis

All athletes in all age groups competing in both the ‘Jungfrau Marathon’ and the ‘Lausanne Marathon’ were considered for analysis by 5-year age groups from 18–25 to 75–79 years. Trends in participation and in the men-to-women ratio across calendar years and across age groups were analyzed using single linear regression analyses. To analyze changes in performance, a mixed-effects regression model with finisher as random variable to consider finishers who completed several races was used. We included sex and calendar year as fixed variables. Absolute sex difference was calculated using the equation ([absolute race time in women] − [absolute race time in men]/[absolute race time in men] × 100). Changes in sex differences across calendar years and were investigated using linear regression models. Statistical analyses were performed using IBM SPSS Statistics (Version 22, IBM SPSS, Chicago, IL, USA). Significance was accepted at P < 0.05 (two-sided for *t* tests). Data in the text and tables are given as mean ± standard deviation (SD).

## Results

### Participation trends

In the ‘Jungfrau Marathon’, participation in all female and male age groups increased with exception of women in age group 18–24 years and men in age groups 30–34, 40–44 and 55–59 years where participation remained unchanged (Table 1). In the ‘Lausanne Marathon’, participation increased in women only in age groups 30–34 to 40–44 years. In men, participation increased in age groups 25–29 to 44–44 years and 60–64 years (Table 1).

**Table 1:** Participation in women and men in age groups in the mountain (upper two panels) and the city marathon (lower two panels)

Sex	Age group	2000	2001	2002	2003	2004	2005	2006	2007	2008	2009	2010	2011	2012	2013	2014	r^2^	p
Jungfrau Marathon																		
Women	18–24	4	9	22	12	15	11	11	12	10	8	12	14	27	23	13	0.18	0.109
	25–29	28	19	39	45	41	47	37	48	37	44	46	46	86	62	60	0.56	0.0012
	30–34	62	62	78	67	75	71	82	92	69	58	66	97	141	97	96	0.37	0.015
	35–39	70	85	109	97	130	114	103	120	114	106	91	94	168	129	116	0.27	0.046
	40–44	95	87	137	112	122	167	162	199	171	198	173	157	279	172	170	0.52	0.002
	45–49	63	66	125	91	109	120	135	162	153	177	168	174	326	178	187	0.64	0.0003
	50–54	47	43	42	43	48	64	67	80	76	102	110	104	231	117	121	0.61	0.005
	55–59	25	25	11	32	25	34	41	34	29	61	35	52	123	57	52	0.44	0.006
	60–64	10	12	7	12	11	16	13	20	19	19	22	22	43	22	29	0.66	0.0002
	65–69	1	3		5	2	2	3	5	5	5	5	3	18	6	8	0.39	0.012
	70–74	2	1				1			1		1	2	6	4	5	0.41	0.0096
Men	18–24	33	42	14	43	65	72	64	75	48	54	56	60	94	68	56	0.34	0.020
	25–29	120	127	79	123	136	114	123	143	135	128	128	128	230	156	190	0.45	0.0061
	30–34	336	330	163	279	335	272	288	317	257	252	245	285	417	268	286	0.004	0.816
	35–39	513	524	313	526	540	496	463	458	430	418	380	365	513	348	324	0.29	0.035
	40–44	525	535	383	562	623	670	665	739	680	697	617	586	877	504	445	0.05	0.384
	45–49	445	445	361	485	539	550	576	637	645	645	648	687	993	602	608	0.53	0.0019
	50–54	335	322	452	346	393	409	437	450	444	509	493	445	894	504	530	0.43	0.0074
	55–59	182	203	251	205	194	238	223	274	255	281	282	294	463	288	289	0.52	0.0023
	60–64	134	117	148	112	108	106	97	133	129	135	126	139	245	135	150	0.19	0.096
	65–69	36	36	50	34	45	53	54	51	39	56	59	49	111	60	48	0.26	0.035
	70–74	8	4	6	3	7	8	5	8	11	14	11	10	27	18	23	0.65	0.0003
	75–79			1	1	1	1	1	2	1	1	1	1	3	2	4	0.58	0.0009
Lausanne Marathon																		
Women	18–24	11	24	8	13	13	17	15	6	6	4	10	7	10	17	15	0.04	0.44
	25–29	20	58	33	23	25	43	27	18	20	21	14	19	29	19	19	0.25	0.055
	30–34	34	60	45	38	30	45	40	32	20	31	13	22	36	29	33	0.31	0.028
	35–39	56	80	51	54	50	60	47	28	31	33	25	32	35	36	25	0.65	0.0002
	40–44	58	61	65	55	65	64	77	44	32	42	34	46	42	33	38	0.52	0.0021
	45–49	26	55	41	45	35	67	47	33	29	39	35	62	29	38	28	0.03	0.48
	50–54	24	34	16	32	25	22	30	25	16	17	27	21	22	36	17	0.02	0.54
	55–59	6	13	9	13	19	14	11	17	13	10	12	16	15	13	15	0.14	0.16
	60–64	4	6	0	2	8		4	17	4	5	8	8	4	8	6	0.09	0.26
	65–69	1	3	1	1	2			8	2	1	2		1	3	3	0.01	0.69
	70–74					1			1					1			0.005	0.78
	75–79		1												1		1.01	1.0
Men	18–24	50	55	43	36	28	34	34	33	27	41	42	36	57	56	43	0.01	0.66
	25–29	140	155	97	114	111	84	95	84	74	85	93	71	82	109	93	0.39	0.012
	30–34	225	259	225	198	217	155	178	139	112	153	122	120	129	139	110	0.78	<0.0001
	35–39	311	318	314	285	284	263	209	210	158	192	209	192	141	173	140	0.87	<0.0001
	40–44	278	305	275	294	325	336	295	267	213	225	223	214	217	222	182	0.65	<0.0001
	45–49	209	188	186	197	233	263	227	183	164	159	214	186	172	188	153	0.19	0.097
	50–54	138	167	150	156	148	170	148	126	116	112	139	103	109	118	125	0.53	0.0019
	55–59	73	71	74	76	91	97	74	87	67	57	66	65	59	62	68	0.26	0.050
	60–64	40	39	48	19	41	37	41	38	30	34	38	49	34	41	33	0.0008	0.91
	65–69	15	14	15	8	11	12	14	17	12	12	11	13	7	18	13	0.004	0.81
	70–74	3	4	2	3	4	6	2	5	4	1	5	7	4	3	5	0.08	0.29
	75–79		1			1	1	1	1	2	1	1	1		2		0.06	0.34

Table 2 summarizes the trends in the men-to-women ratio across age groups and across calendar years for both the ‘Jungfrau Marathon’ and the ‘Lausanne Marathon’. In the ‘Jungfrau Marathon’, the men-to-women ratio increased across age groups in 2008, 2010, and 2012–2014. Across calendar years, the ratio decreased in age groups 30–34 to 50–54 and 60–64 to 65–69 years. For the ‘Lausanne Marathon’, the men-to-women ratio increased across age groups in 2000, 2002, 2003, 2005, 2006 and 2008. Across calendar years, the ratio decreased in age group 55–59 years and remained unchanged for all other age groups.

**Table 2:** The men-to-women ratio for the mountain and the city marathon across age groups and calendar years

Age group	2000	2001	2002	2003	2004	2005	2006	2007	2008	2009	2010	2011	2012	2013	2014	r^2^	p
Jungfrau Marathon																	
18–24	8.2	4.6	0.6	3.5	4.3	6.5	5.8	6.2	4.8	6.7	4.6	4.2	3.4	2.9	4.3	0.03	0.52
25–29	4.2	6.6	2.0	2.7	3.3	2.4	3.3	2.9	3.6	2.9	2.7	2.7	2.6	2.5	3.1	0.19	0.10
30–34	5.4	5.3	2.0	4.1	4.4	3.8	3.5	3.4	3.7	4.3	3.7	2.9	2.9	2.7	2.9	0.34	0.02
35–39	7.3	6.1	2.8	5.4	4.1	4.3	4.4	3.8	3.7	3.9	4.1	3.8	3.0	2.6	2.7	0.54	0.001
40–44	5.5	6.1	2.7	5.0	5.1	4.0	4.1	3.7	3.9	3.5	3.5	3.7	3.1	2.9	2.6	0.57	0.001
45–49	7.0	6.7	2.8	5.3	4.9	4.5	4.2	3.9	4.2	3.6	3.8	3.9	3.0	3.3	3.2	0.53	0.002
50–54	7.1	7.4	10.7	8.0	8.1	6.3	6.5	5.6	5.8	4.9	4.4	4.2	3.8	4.3	4.3	0.73	<0.0001
55–59	7.2	8.1	22.8	6.4	7.7	7.0	5.4	8.0	8.7	4.6	8.0	5.6	3.7	5.0	5.5	0.21	0.88
60–64	13.4	9.7	21.1	9.3	9.8	6.6	7.4	6.6	6.7	7.1	5.7	6.3	5.6	6.1	5.1	0.49	0.003
65–69	36.0	12.0		6.8	22.5	26.5	18.0	10.2	7.8	11.2	11.8	16.3	6.1	10.0	6.0	0.35	0.02
70–74	4.0	4.0				8.0			11.0		11.0	5.0	4.5	4.5	4.6	0.00	0.85
r^2^	0.19	0.24	0.12	0.05	0.18	0.30	0.09	0.04	0.71	0.01	0.64	0.33	0.61	0.54	0.46		
p	0.18	0.13	0.30	0.49	0.19	0.08	0.36	0.56	0.001	0.73	0.002	0.06	0.004	0.01	0.02		
Lausanne Marathon																	
18–24	4.5	2.2	5.3	2.7	2.1	2.0	2.2	5.5	4.5	10.2	4.2	5.1	5.7	3.2	2.8	0.05	0.41
25–29	7.0	2.6	2.9	4.9	4.4	1.9	3.5	4.6	3.7	4.0	6.6	3.7	2.8	5.7	4.8	0.01	0.71
30–34	6.6	4.3	5.0	5.2	7.2	3.4	4.4	4.3	5.6	4.9	9.3	5.4	3.5	4.7	3.3	0.02	0.57
35–39	5.5	3.9	6.1	5.2	5.6	4.3	4.4	7.5	5.0	5.8	8.3	6.0	4.0	4.8	5.6	0.01	0.66
40–44	4.7	5.0	4.2	5.3	5.0	5.2	3.8	6.0	6.6	5.3	6.5	4.6	5.1	6.7	4.7	0.14	0.17
45–49	8.0	3.4	4.5	4.3	6.6	3.9	4.8	5.5	5.6	4.0	6.1	3.0	5.9	4.9	5.4	0.01	0.73
50–54	5.7	4.9	9.3	4.8	5.9	7.7	4.9	5.0	7.2	6.5	5.1	4.9	4.9	3.2	7.3	0.06	0.39
55–59	12.1	5.4	8.2	5.8	4.7	6.9	6.7	5.1	5.1	5.7	5.5	4.0	3.9	4.7	4.5	0.46	0.005
60–64	10.0	6.5		9.5	5.1		10.2	2.2	7.5	6.8	4.7	6.1	8.5	5.1	5.5	0.14	0.20
65–69	15.0	4.6	15.0	8.0	5.5			2.1	6.0	12.0	5.5		7.0	6.0	4.3	0.20	0.13
70–74					4.0			5.0					4.0			0.02	0.90
75–79		1.0												2.0			
r^2^	0.61	0.02	0.65	0.60	0.02	0.83	0.73	0.23	0.45	0.08	0.12	0.00	0.17	0.04	0.19		
p	0.007	0.64	0.008	0.008	0.66	0.001	0.003	0.13	0.03	0.42	0.31	0.91	0.20	0.53	0.20		

### Performance trends

Table 3 presents the race times in the ‘Jungfrau Marathon’ and the ‘Lausanne Marathon’ for women and men by age group. In the ‘Jungfrau Marathon’, race time showed a significant and positive change for sex for all age groups (i.e. the runners became slower across age groups) with the exception of age group 75–79 years (Table 4). For calendar year, the changes were significant and positive (i.e. the runners became slower across years) for age groups 18–24 to 70–74 years. In the ‘Lausanne Marathon’, race time showed a significant and positive change (i.e. the runners became slower across age groups) for sex for all age groups from 18–24 to 70–74 years (Table 5). For calendar year, the changes were significant and positive (i.e. the runners became slower across years) for age groups 18–24 and 30–34 to 65–69 years, but not for 25–29, 70–74 and 75–79 years.

**Table 3:** Performance in women and men in age groups in the mountain (upper two panels) and the city marathon (lower two panels)

Sex	Age group	2000	2001	2002	2003	2004	2005	2006	2007	2008	2009	2010	2011	2012	2013	2014
Jungfrau Marathon																
Women	18–24	5:00 ± 0:49	4:53 ± 0:48	5:25 ± 0:37	5:23 ± 0:38	5:38 ± 0:42	5:21 ± 0:35	5:27 ± 0:40	4:57 ± 0:32	5:20 ± 0:39	5:18 ± 0:32	5:19 ± 0:41	5:51 ± 0:32	5:31 ± 0:47	5:19 ± 0:32	5:50 ± 0:41
	25–29	4:52 ± 0:38	4:51 ± 0:47	5:08 ± 0:41	5:06 ± 0:44	5:02 ± 0:34	5:06 ± 0:36	5:17 ± 0:47	5:06 ± 0:42	5:15 ± 0:41	5:06 ± 0:34	5:13 ± 0:38	5:26 ± 0:36	5:17 ± 0:51	5:17 ± 0:36	5:29 ± 0:34
	30–34	5:02 ± 0:37	5:00 ± 0:37	5:12 ± 0:35	5:08 ± 0:41	5:14 ± 0:41	5:06 ± 0:37	5:10 ± 0:35	5:15 ± 0:44	5:12 ± 0:39	5:12 ± 0:41	5:06 ± 0:41	5:24 ± 0:49	5:26 ± 0:47	5:19 ± 0:46	5:22 ± 0:42
	35–39	4:59 ± 0:41	5:01 ± 0:35	5:17 ± 0:33	5:13 ± 0:31	5:17 ± 0:36	5:19 ± 0:38	5:17 ± 0:39	5:08 ± 0:43	5:19 ± 0:37	5:15 ± 0:44	5:17 ± 0:43	5:17 ± 0:40	5:16 ± 0:45	5:21 ± 0:41	5:16 ± 0:42
	40–44	5:19 ± 0:36	5:12 ± 0:31	5:14 ± 0:35	5:16 ± 0:39	5:22 ± 0:33	5:26 ± 0:32	5:27 ± 0:35	5:24 ± 0:38	5:25 ± 0:35	5:19 ± 0:37	5:26 ± 0:36	5:29 ± 0:36	5:27 ± 0:39	5:26 ± 0:37	5:26 ± 0:36
	45–49	5:23 ± 0:37	5:17 ± 0:28	5:25 ± 0:31	5:26 ± 0:29	5:34 ± 0:32	5:33 ± 0:32	5:34 ± 0:37	5:33 ± 0:36	5:27 ± 0:34	5:31 ± 0:34	5:28 ± 0:34	5:34 ± 0:36	5:38 ± 0:36	5:33 ± 0:34	5:37 ± 0:32
	50–54	5:35 ± 0:31	5:35 ± 0:31	5:41 ± 0:25	5:35 ± 0:30	5:38 ± 0:34	5:37 ± 0:33	5:36 ± 0:28	5:42 ± 0:41	5:37 ± 0:32	5:45 ± 0:31	5:40 ± 0:31	5:47 ± 0:28	5:42 ± 0:36	5:45 ± 0:34	5:42 ± 0:32
	55–59	5:30 ± 0:33	5:37 ± 0:43	5:56 ± 0:25	5:40 ± 0:31	5:46 ± 0:29	5:51 ± 0:29	5:47 ± 0:31	5:53 ± 0:29	5:56 ± 0:30	5:51 ± 0:31	5:44 ± 0:30	5:49 ± 0:31	5:57 ± 0:35	5:45 ± 0:33	5:53 ± 0:31
	60–64	5:38 ± 0:35	5:26 ± 0:28	5:38 ± 0:16	5:36 ± 0:32	5:42 ± 0:37	5:48 ± 0:25	5:56 ± 0:24	5:57 ± 0:25	5:56 ± 0:20	6:16 ± 0:13	6:00 ± 0:29	6:04 ± 0:28	6:11 ± 0:30	6:01 ± 0:29	6:01 ± 0:33
	65–69	5:56	5:43 ± 0:22		5:58 ± 0:15	5:57 ± 0:02	5:50 ± 0:41	5:56 ± 0:40	5:49 ± 0:24	5:40 ± 0:21	5:39 ± 0:41	5:56 ± 0:47	6:31 ± 0:11	6:26 ± 0:15	6:04 ± 0:17	6:01 ± 0:32
	70–74	6:10 ± 0:19	6:28				5:57			6:17		5:36	6:19 ± 0:28	6:09 ± 0:34	6:04 ± 0:35	6:18 ± 0:28
Men	18–24	4:37 ± 0:36	4:36 ± 0:32	5:02 ± 0:36	4:58 ± 0:34	5:04 ± 0:38	4:98 ± 0:40	5:13 ± 0:41	5:09 ± 0:45	5:11 ± 0:37	5:02 ± 0:44	4:51 ± 0:46	5:09 ± 0:48	5:09 ± 0:42	5:08 ± 0:43	5:18 ± 0:43
	25–29	4:40 ± 0:40	4:37 ± 0:43	4:58 ± 0:35	4:52 ± 0:40	4:54 ± 0:41	4:55 ± 0:41	4:58 ± 0:39	4:58 ± 0:49	4:59 ± 0:44	4:56 ± 0:45	4:56 ± 0:44	5:03 ± 0:45	5:01 ± 0:48	4:56 ± 0:45	4:58 ± 0:43
	30–34	4:38 ± 0:41	4:37 ± 0:41	4:54 ± 0:38	4:55 ± 0:42	4:58 ± 0:44	4:56 ± 0:42	4:57 ± 0:42	4:54 ± 0:43	4:59 ± 0:39	4:51 ± 0:44	4:55 ± 0:48	5:02 ± 0:43	4:57 ± 0:45	4:56 ± 0:42	4:55 ± 0:43
	35–39	4:40 ± 0:39	4:40 ± 0:41	4:57 ± 0:38	4:54 ± 0:40	4:59 ± 0:41	4:54 ± 0:40	5:00 ± 0:44	4:53 ± 0:44	5:02 ± 0:41	4:56 ± 0:42	4:56 ± 0:41	5:03 ± 0:42	5:06 ± 0:43	5:01 ± 0:43	4:59 ± 0:47
	40–44	4:44 ± 0:35	4:44 ± 0:37	5:00 ± 0:34	4:57 ± 0:37	5:03 ± 0:39	5:02 ± 0:38	5:06 ± 0:40	5:05 ± 0:41	5:05 ± 0:37	5:03 ± 0:40	5:04 ± 0:41	5:13 ± 0:42	5:11 ± 0:41	5:09 ± 0:41	5:06 ± 0:40
	45–49	4:55 ± 0:36	4:50 ± 0:36	5:05 ± 0:35	5:03 ± 0:36	5:09 ± 0:38	5:05 ± 0:38	5:09 ± 0:36	5:11 ± 0:37	5:12 ± 0:38	5:07 ± 0:39	5:11 ± 0:39	5:13 ± 0:39	5:14 ± 0:39	5:11 ± 0:38	5:10 ± 0:42
	50–54	5:02 ± 0:36	4:57 ± 0:34	5:09 ± 0:37	5:14 ± 0:36	5:21 ± 0:38	5:18 ± 0:36	5:21 ± 0:36	5:17 ± 0:41	5:20 ± 0:35	5:16 ± 0:37	5:18 ± 0:38	5:23 ± 0:39	5:24 ± 0:40	5:19 ± 0:37	5:19 ± 0:41
	55–59	5:13 ± 0:35	5:11 ± 0:36	5:21 ± 0:37	5:26 ± 0:35	5:29 ± 0:37	5:25 ± 0:38	5:27 ± 0:37	5:29 ± 0:37	5:27 ± 0:35	5:25 ± 0:35	5:31 ± 0:37	5:36 ± 0:37	5:34 ± 0:38	5:34 ± 0:38	5:30 ± 0:38
	60–64	5:27 ± 0:41	5:21 ± 0:36	5:27 ± 0:35	5:32 ± 0:35	5:36 ± 0:36	5:37 ± 0:33	5:43 ± 0:31	5:40 ± 0:33	5:38 ± 0:35	5:38 ± 0:33	5:38 ± 0:36	5:45 ± 0:37	5:45 ± 0:40	5:34 ± 0:34	5:35 ± 0:45
	65–69	5:31 ± 0:40	5:25 ± 0:30	5:34 ± 0:35	5:36 ± 0:30	5:44 ± 0:31	5:41 ± 0:31	5:45 ± 0:33	5:40 ± 0:37	5:59 ± 0:28	5:46 ± 0:31	5:51 ± 0:32	5:52 ± 0:29	5:59 ± 0:37	5:44 ± 0:34	5:32 ± 0:41
	70–74	6:08 ± 0:32	5:41 ± 0:44	6:03 ± 0:28	5:25 ± 0:04	5:45 ± 0:25	5:58 ± 0:28	5:59 ± 0:31	5:59 ± 0:30	5:52 ± 0:29	5:57 ± 0:27	5:49 ± 0:32	5:53 ± 0:30	5:10 ± 0:40	5:52 ± 0:27	5:53 ± 0:34
	75–79			5:44	6:08	6:13	6:16	5:56	5:49 ± 0:18	6:18	6:29	5:29	5:44	5:55 ± 0:28	6:21 ± 0:07	5:30 ± 0:56
Lausanne Marathon																
Women	18–24	4:14 ± 0:42	4:43 ± 0:57	3:40 ± 0:38	3:54 ± 0:38	3:52 ± 1:03	4:27 ± 0:44	4:12 ± 0:37	3:42 ± 0:43	3:36 ± 0:41	4:22 ± 0:24	4:11 ± 0:42	4:07 ± 1:02	3:50 ± 0:22	4:58 ± 1:02	4:25 ± 0:35
	25–29	4:03 ± 0:38	4:33 ± 0:47	3:50 ± 0:37	3:57 ± 0:41	4:09 ± 0:29	4:24 ± 0:58	4:12 ± 0:42	4:15 ± 0:37	4:12 ± 0:34	3:51 ± 0:19	3:57 ± 0:27	4:15 ± 0:42	4:06 ± 0:28	3:52 ± 0:25	4:18 ± 0:48
	30–34	3:57 ± 0:27	4:25 ± 0:40	3:55 ± 0:33	4:05 ± 0:37	4:07 ± 0:37	4:22 ± 0:44	4:02 ± 0:29	4:03 ± 0:36	4:14 ± 0:28	4:00 ± 0:30	3:58 ± 0:26	4:03 ± 0:39	4:17 ± 0:37	4:07 ± 0:36	4:13 ± 0:33
	35–39	3:55 ± 0:35	3:56 ± 0:46	4:03 ± 0:33	4:04 ± 0:26	4:07 ± 0:30	4:03 ± 0:30	4:05 ± 0:35	3:50 ± 0:35	3:54 ± 0:24	4:00 ± 0:27	4:00 ± 0:38	4:05 ± 0:31	4:14 ± 0:36	4:09 ± 0:39	4:11 ± 0:40
	40–44	3:59 ± 0:29	4:18 ± 0:50	4:03 ± 0:32	4:01 ± 0:32	4:11 ± 0:30	4:12 ± 0:35	4:20 ± 0:31	4:10 ± 0:29	4:05 ± 0:29	4:07 ± 0:38	4:04 ± 0:34	4:09 ± 0:38	4:15 ± 0:46	4:10 ± 0:32	4:03 ± 0:34
	45–49	4:17 ± 0:37	4:49 ± 0:57	4:15 ± 0:24	4:22 ± 0:39	4:09 ± 0:30	4:28 ± 0:52	4:18 ± 0:41	4:15 ± 0:31	4:04 ± 0:29	4:06 ± 0:35	4:19 ± 0:37	4:12 ± 0:34	4:18 ± 0:35	4:21 ± 0:37	4:15 ± 0:25
	50–54	4:26 ± 0:40	4:44 ± 0:56	4:04 ± 0:19	4:15 ± 0:32	4:25 ± 0:24	4:46 ± 1:10	4:31 ± 0:39	4:33 ± 0:39	4:09 ± 0:28	4:33 ± 0:39	4:15 ± 0:31	4:12 ± 0:36	4:17 ± 0:42	4:23 ± 0:35	4:15 ± 0:32
	55–59	4:38 ± 0:29	4:36 ± 0:57	4:09 ± 0:39	4:22 ± 0:49	4:29 ± 0:34	5:11 ± 1:42	4:30 ± 0:29	4:59 ± 0:51	4:44 ± 0:32	4:17 ± 0:39	4:24 ± 0:39	4:44 ± 0:41	4:17 ± 0:26	4:29 ± 0:30	4:26 ± 0:33
	60–64	4:54 ± 0:36	4:54 ± 0:59		5:18 ± 0:36	4:56 ± 0:33		4:21 ± 0:31	5:13 ± 0:41	4:00 ± 0:28	5:20 ± 0:40	5:12 ± 0:48	4:38 ± 0:30	4:55 ± 1:06	4:50 ± 0:26	5:24 ± 0:44
	65–69	5:05	4:45 ± 0:41	3:54	4:41	5:20 ± 1:17			5:42 ± 0:30	5:47 ± 0:27	5:53	4:21 ± 0:32		5:26	5:32 ± 0:14	5:07 ± 0:35
	70–74					5:48			6:19					6:17		
	75–79		6:46												5:20	
Men	18–24	3:46 ± 0:42	3:40 ± 0:42	3:27 ± 0:30	3:57 ± 0:36	3:49 ± 0:39	3:40 ± 0:34	3:50 ± 0:38	3:54 ± 0:39	3:59 ± 0:42	3:53 ± 0:33	3:56 ± 0:38	3:50 ± 0:33	3:53 ± 0:27	4:06 ± 0:51	3:51 ± 0:26
	25–29	3:39 ± 0:38	3:45 ± 0:39	3:35 ± 0:34	3:38 ± 0:36	3:46 ± 0:28	3:37 ± 0:30	3:47 ± 0:38	3:46 ± 0:40	3:43 ± 0:31	3:50 ± 0:37	3:46 ± 0:35	3:45 ± 0:29	3:45 ± 0:31	3:44 ± 0:41	3:52 ± 0:36
	30–34	3:36 ± 0:33	3:42 ± 0:36	3:39 ± 0:35	3:36 ± 0:36	3:45 ± 0:37	3:35 ± 0:32	3:43 ± 0:33	3:37 ± 0:30	3:41 ± 0:34	3:44 ± 0:31	3:44 ± 0:33	3:44 ± 0:34	3:51 ± 0:37	3:44 ± 0:36	3:50 ± 0:35
	35–39	3:39 ± 0:30	3:38 ± 0:31	3:35 ± 0:30	3:36 ± 0:30	3:45 ± 0:32	3:40 ± 0:32	3:47 ± 0:34	3:42 ± 0:32	3:45 ± 0:35	3:44 ± 0:30	3:45 ± 0:28	3:46 ± 0:34	3:48 ± 0:29	3:52 ± 0:31	3:48 ± 0:36
	40–44	3:40 ± 0:32	3:45 ± 0:34	3:38 ± 0:32	3:44 ± 0:32	3:49 ± 0:31	3:41 ± 0:29	3:49 ± 0:33	3:46 ± 0:32	3:44 ± 0:30	3:43 ± 0:31	3:49 ± 0:33	3:51 ± 0:33	3:49 ± 0:31	3:45 ± 0:33	3:50 ± 0:32
	45–49	3:48 ± 0:31	3:45 ± 0:29	3:44 ± 0:27	3:46 ± 0:30	3:55 ± 0:33	3:48 ± 0:33	3:57 ± 0:33	3:52 ± 0:38	3:49 ± 0:30	3:51 ± 0:31	3:46 ± 0:30	3:54 ± 0:30	3:53 ± 0:32	3:53 ± 0:32	3:57 ± 0:34
	50–54	3:53 ± 0:33	3:54 ± 0:32	3:51 ± 0:31	3:52 ± 0:32	4:04 ± 0:30	3:52 ± 0:30	4:01 ± 0:30	4:01 ± 0:33	3:56 ± 0:28	3:56 ± 0:34	3:58 ± 0:30	3:58 ± 0:36	4:01 ± 0:36	4:05 ± 0:35	4:03 ± 0:38
	55–59	3:57 ± 0:38	4:00 ± 0:29	4:01 ± 0:35	3:59 ± 0:32	4:13 ± 0:32	3:58 ± 0:26	4:11 ± 0:34	4:06 ± 0:36	4:07 ± 0:34	4:03 ± 0:33	4:03 ± 0:31	4:09 ± 0:37	4:07 ± 0:30	4:09 ± 0:31	4:07 ± 0:39
	60–64	4:21 ± 0:43	4:26 ± 0:40	4:26 ± 0:38	4:13 ± 0:31	4:08 ± 0:28	4:04 ± 0:39	4:23 ± 0:41	4:21 ± 0:43	4:20 ± 0:31	4:16 ± 0:41	4:15 ± 0:33	4:18 ± 0:40	4:25 ± 0:41	4:24 ± 0:33	4:18 ± 0:30
	65–69	4:16 ± 0:37	4:38 ± 0:56	4:32 ± 0:25	4:14 ± 0:35	4:48 ± 0:37	4:19 ± 0:25	4:47 ± 0:33	4:24 ± 0:39	4:30 ± 0:27	4:28 ± 0:25	4:18 ± 0:29	4:37 ± 0:29	4:26 ± 0:24	4:48 ± 0:56	4:29 ± 0:38
	70–74	4:34 ± 0:43	4:22 ± 0:07	4:25 ± 0:15	3:49 ± 0:16	4:36 ± 0:13	4:30 ± 0:36	5:00 ± 0:36	5:16 ± 0:37	4:51 ± 0:14	4:01	5:05 ± 0:54	4:36 ± 0:42	4:31 ± 0:35	4:04 ± 0:12	4:31 ± 0:38
	75–79		4:26			5:50	4:14	5:35	5:32	5:10 ± 0:55	5:24	5:45	5:55		5:28 ± 0:25

**Table 4:** Results of the mixed effects regression analyses for race time in the ‘Jungfrau Marathon’

Parameter	Estimate	Standard error	*df*	t	*p* value
18–24					
Constant term	−1468.67	656.37	984.51	−2.23	0.025
Female sex	20.32	3.67	755.22	5.53	<0.0001
Calendar year	0.88	0.32	984.55	2.70	0.007
25–29					
Constant term	−1627.79	401.69	2542.02	−4.05	<0.0001
Female sex	18.26	2.17	2073.43	8.38	<0.0001
Calendar year	0.95	0.20	2542.07	4.79	<0.0001
30–34					
Constant term	−1764.41	270.53	5350.93	−6.52	<0.0001
Female sex	20.86	1.57	4117.81	13.21	<0.0001
Calendar year	1.02	0.13	5350.98	7.61	<0.0001
35–39					
Constant term	−1880.69	222.38	8062.32	−8.45	<0.0001
Female sex	19.38	1.35	5718.30	14.33	<0.0001
Calendar year	1.08	0.11	8062.38	9.79	<0.0001
40–44					
Constant term	−2350.94	183.75	11,098.30	−12.79	<0.0001
Female sex	20.53	1.06	7616.11	19.20	<0.0001
Calendar year	1.32	0.09	11,098.40	14.45	<0.0001
45–49					
Constant term	−2008.87	181.75	10,676.10	−11.05	<0.0001
Female sex	23.67	1.07	7201.81	21.96	<0.0001
Calendar year	1.15	0.09	10,676.17	12.76	<0.0001
54–54					
Constant term	−2461.63	202.63	8035.83	−12.14	<0.0001
Female sex	22.71	1.33	5336.54	16.96	<0.0001
Calendar year	1.38	0.10	8035.96	13.72	<0.0001
55–59					
Constant term	−3145.87	273.07	4263.96	−11.52	<0.0001
Female sex	21.21	1.85	2894.57	11.46	<0.0001
Calendar year	1.73	0.13	4264.08	12.72	<0.0001
60–64					
Constant term	−3685.21	374.10	2154.69	−9.85	<0.0001
Female sex	17.95	2.92	1363.42	6.14	<0.0001
Calendar year	2.00	0.18	2154.72	10.75	<0.0001
65–69					
Constant term	−3880.01	616.55	794.88	−6.29	<0.0001
Female sex	18.38	5.31	493.15	3.46	0.001
Calendar year	2.10	0.30	794.89	6.85	<0.0001
70–74					
Constant term	−2739.43	1248.46	156.09	−2.19	0.030
Female sex	6.60	8.59	100.46	0.76	0.444
Calendar year	1.54	0.62	156.09	2.48	0.014
75–79					
Constant term	3023.71	4920.13	11.05	0.61	0.551
Calendar year	−1.32	2.44	11.06	−0.54	0.598

**Table 5:** Results of the mixed effects regression analyses for race time in the ‘Lausanne Marathon’

Parameter	Estimate	Standard error	*df*	t	p value
18–24					
Constant term	−1671.42	652.21	775.44	−2.56	0.011
Female sex	25.36	3.65	729.37	6.94	<0.0001
Calendar year	0.94	0.32	775.44	2.91	0.004
25–29					
Constant term	−54.73	399.06	1839.49	−0.13	0.891
Female sex	26.47	2.23	1660.61	11.86	<0.0001
Calendar year	0.13	0.19	1839.50	0.70	0.483
30–34					
Constant term	−556.59	305.88	2861.85	−1.82	0.069
Female sex	26.87	1.79	2595.44	15.00	<0.0001
Calendar year	0.38	0.15	2861.88	2.54	0.011
35–39					
Constant term	−1098.15	247.61	3963.50	−4.43	<0.0001
Female sex	23.05	1.48	3494.68	15.49	<0.0001
Calendar year	0.65	0.12	3963.51	5.33	<0.0001
40–44					
Constant term	−703.52	241.15	4445.91	−2.91	0.004
Female sex	24.70	1.40	3881.69	17.53	<0.0001
Calendar year	0.46	0.12	4445.94	3.85	<0.0001
45–49					
Constant term	−546.72	278.20	3485.36	−1.96	0.049
Female sex	29.93	1.61	2969.98	18.53	<0.0001
Calendar year	0.38	0.13	3485.35	2.79	0.005
50–54					
Constant term	−771.09	333.87	2366.70	−2.31	0.021
Female sex	28.46	2.08	2005.98	13.67	<0.0001
Calendar year	0.50	0.16	2366.72	3.02	0.003
55–59					
Constant term	−1001.66	493.28	1277.73	−2.03	0.043
Female sex	31.87	3.07	1026.03	10.36	<0.0001
Calendar year	0.62	0.24	1277.73	2.52	0.012
60–64					
Constant term	−1639.41	705.31	643.37	−2.32	0.020
Female sex	44.02	4.73	542.93	9.28	<0.0001
Calendar year	0.94	0.35	643.37	2.68	0.007
65–69					
Constant term	−2890.45	1215.31	217.99	−2.37	0.018
Female sex	43.60	8.10	178.72	5.38	<0.0001
Calendar year	1.57	0.60	217.99	2.60	0.010
70–74					
Constant term	−2691.53	2308.33	56.65	−1.16	0.248
Female sex	89.32	21.71	53.54	4.11	<0.0001
Calendar year	1.47	1.14	56.65	1.28	0.203
75–79					
Constant term	−669.16	4482.108462	13.40	−0.14	0.884
Female sex	43.69	26.92	9.86	1.62	0.136
Calendar year	0.49	2.23	13.40	0.22	0.829

### Sex difference

In the ‘Jungfrau Marathon’, the sex difference increased in age groups 25–29 (from 4 to 10 %) and 60–64 years (from 3 to 8 %) but decreased in age group 40–44 years (from 12 to 6 %) (Table 6). For age groups 18–24, 30–34, 35–39, 45–49, 50–54, 55–59, 60–64, 65–69, and 70–74 years, the values were 7.3 ± 2.9, 6.3 ± 2.0, 6.2 ± 1.3, 7.5 ± 1.3, 7.5 ± 2.1, 6.4 ± 2.2, 5.2 ± 2.9, and 6.0 ± 4.5 %, respectively. In the ‘Lausanne Marathon’, however, the sex difference showed no changes. The values for the sex difference were 10.4 ± 8.1, 10.6 ± 5.5, 11.2 ± 4.5, 9.1 ± 3.5, 10.2 ± 2.7, 12.1 ± 5.7, 10.7 ± 5.8, 11.7 ± 7.2, 16.0 ± 8.1, 28.3 ± 9.7, and 27.5 ± 35.1 % for age groups 18–24, 25–29, 30–34, 35–39, 40–44, 45–49, 50–54, 55–59, 60–64, and 65–69, respectively (Table 6).

**Table 6:** Sex difference (%) in age groups in the mountain (upper two panels) and the city marathon (lower two panels)

Age group	2000	2001	2002	2003	2004	2005	2006	2007	2008	2009	2010	2011	2012	2013	2014	r^2^	p
Jungfrau Marathon																	
18–24	8	6	8	8	11	8	5	4	3	5	10	13	7	4	10	0.001	0.906
25–29	4	5	3	5	3	4	6	3	5	4	6	8	5	7	10	0.463	0.005
30–34	9	8	6	5	5	3	4	7	4	7	4	7	9	8	9	0.047	0.436
35–39	7	8	6	7	6	8	6	5	6	6	7	5	3	7	6	0.218	0.079
40–44	12	10	4	6	6	8	7	6	7	5	7	5	5	6	6	0.278	0.043
45–49	9	9	6	8	8	9	8	7	5	8	6	6	8	7	9	0.066	0.354
50–54	11	12	10	7	5	6	5	8	5	9	7	7	6	8	7	0.195	0.098
55–59	5	8	11	4	5	8	6	7	9	8	4	4	7	3	7	0.066	0.353
60–64	3	2	3	1	2	3	4	5	5	8	7	6	8	8	8	0.820	<0.0001
65–69	7	6		7	4	2	3	3	5	2	1	11	8	6	9	0.046	0.457
70–74	1	14									4	7		3	7	0.024	0.685
Lausanne Marathon																	
18–24	12	29	6	1	1	21	10	5	10	12	6	7	1	21	14	0.005	0.792
25–29	11	21	7	9	10	21	11	13	13	1	5	13	9	4	11	0.141	0.166
30–34	10	19	7	13	10	22	8	12	15	7	6	8	11	10	10	0.099	0.252
35–39	8	17	13	13	10	10	8	4	4	7	7	8	11	7	10	0.192	0.102
40–44	9	15	11	8	10	14	14	11	9	11	7	8	11	11	5	0.176	0.119
45–49	13	28	14	16	6	18	9	10	7	7	15	8	11	12	8	0.241	0.063
50–54	14	21	6	10	8	23	12	14	6	16	7	6	6	7	5	0.265	0.049
55–59	17	15	4	10	6	30	8	22	15	6	9	14	4	8	8	0.071	0.335
60–64	13	19		26	19		1	19	8	25	23	8	11	10	26	0.002	0.865
65–69	19	2	14	10	11			29	29	31	1		22	15	14	0.051	0.480
70–74					26			20					39			0.590	0.442
75–79		53												2			

## Discussion

The aim of the present study was investigate participation and performance trends in the mountain marathon ‘Jungfrau Marathon’ with the hypothesis that participation would increase and performance would improve as it has been reported for a large city marathon such as the ‘New York City Marathon’. The main findings were for both the ‘Jungfrau Marathon’ and the ‘Lausanne Marathon’ that (1) participation increased in most age groups but (2) performance decreased in most age groups for both women and men.

### Participation trends

City marathons have been held for decades whereas the first official marathon was in the 1896 Summer Olympics (http://www.olympic.org/athens-1896-summer-olympics). Mountain marathon became popular much later (http://www.wmra.info/). The present findings in participation and performance in these mountain marathoners competing in the ‘Jungfrau Marathon’ might be specific for Switzerland. Teutsch et al. (2013) investigated participation and performance trends in age group inline skaters competing between 1998 and 2009 in the longest inline race in Europe, the ‘Inline One-Eleven’ over 111 km, which was also held in Switzerland. During the 12-year period, overall participation increased until 2003 but decreased thereafter. The relative participation in athletes younger than 40 years old decreased while relative participation increased for athletes older than 40 years. The race times of the best female and male skaters stabilized across years (Teutsch et al. 2013). In the 120-km ultra-endurance mountain bike race the ‘Swiss Bike Masters’ held in Switzerland from 1995 to 2009, similar findings were reported (Gloor et al. 2013; Haupt et al. 2013). The number of male finishers decreased while women’s participation has remained low. Performances of the annual fastest women improved, while performances of the annual fastest men remained unchanged (Gloor et al. 2013). However, this trend of an increase in participation with impairment in performance was not obvious in all races held in Switzerland. In ‘Ironman Switzerland’ held between 1995 and 2011, the number of finishers increased the successful finishers were able to improve their race times (Rüst et al. 2012). In the ‘Powerman Zofingen’ long-distance duathlon (10-km run, 150-km cycle, and 30-km run) held from 2002 to 2011 in Switzerland, the participation remained across years fairly stable and similarly the performance in running and cycling times were also fairly for both male and female elite duathletes (Rüst et al. 2013). An increase in participation and a decrease in performance are most probably not specific for races held in Switzerland, although not all mentioned studies investigated the trends for age group athletes. The fact that the participation in the mountain marathon grew but not in the city marathon might be due to the fact that running on concrete surface is associated with increased potential of injury (Van der Worp et al. 2015).

### Performance trends

The finding that age group athletes became slower in the mountain marathon ‘Jungfrau Marathon’ is controversial to reported findings for a large city marathon such as the ‘New York City Marathon’ (Jokl et al. 2004; Lepers and Cattagni 2012). The most likely explanation for this difference in the fact that we included all recorded finishers for each age group for our analysis while Jokl et al. (2004) considered the fastest 50 women and men for each age group and Lepers and Cattagni (2012) the ten fastest women and men. With an increasing participation in the ‘New York City Marathon’ the density of elite runners might become higher and therefore the fastest 10 and fastest 50 finishers became faster. On the other side, an increase of the participation might lower the average performance level. The selection of a fixed number of athletes for each age group (i.e. the ten fastest) may lead to a selection bias. When we consider all successful finishers for each age group, the number of athletes for each age group will increase and also include slow and weak runners who finish just within the time limit. Therefore, the mean marathon race time in an age group with a large number of finishers will be relatively high.

A further interesting finding was that also the race times in the city marathon ‘Lausanne Marathon’ became slower. Similarly to the ‘Jungfrau Marathon’, all finishers were included in each age group. The decrease in performance in both the ‘Jungfrau Marathon’ and the ‘Lausanne Marathon’ could be a specific phenomenon for Swiss endurance races. A recent study investigating all half and full marathons in Switzerland held from 2000 to 2010 showed that that participation increased in half marathons but decreased in full marathons (Anthony et al. 2014). This might be due to the fact that city marathons exist in almost every major European city. Only few marathons around the world are considered ‘World Marathon Majors’ and attract tens of thousands of athletes (http://www.worldmarathonmajors.com). The ‘New York City Marathon’ is one of them (Jokl et al. 2004, Lepers and Cattagni 2012). Most other city marathons attract much less runner. Considering performance, half-marathoners stabilized their race times whereas marathoners improved (Anthony et al. 2014). However, similarly to the studies of Jokl et al. (2004) and Lepers and Cattagni (2012), Anthony et al. (2014) considered the ten fastest of each age group.

These disparate findings for the ‘Jungfrau Marathon’ concerning a decrease in performance compared to the ‘New York City Marathon’ with an increase in performance for age group athletes (Jokl et al. 2004; Lepers and Cattagni 2012) might be explained by the different analyses or by local differences as discussed above. Future studies might investigate participation and performance trends in other mountain marathons or mountain ultra-marathons held in other countries such as Switzerland.

### Sex difference

The sex difference increased in the ‘Jungfrau Marathon’ in age groups 25–29 and 60–64 years but decreased in 40–44 years. In the ‘Lausanne Marathon’, the sex difference showed no changes. Overall, the sex differences were lower in the ‘Jungfrau Marathon’ (~6–7 %) compared to the ‘Lausanne Marathon’ where the sex differences were ~10–12 % from age group 18–24 to 55–59 years. For age groups 60–64 to 70–74 years, the sex difference continuously increased. Similar findings have been reported by Senefeld et al. (2015) for age group marathoners, where the sex difference increased across age groups from 10.6 ± 0.5 % for the 25–29-year-olds to 23.3 ± 2.6 % for the 80–84-year-olds. The increase in sex difference in marathon running with increasing age is most likely due to the lower number of women finishers than men (Hunter and Stevens 2013). Considering our own data, the men-to-women ratio increased in the mountain marathon mainly in the years 2008–2014 whereas in the city marathon, the increase was in earlier years between 2000 and 2008. Obviously, there was a shift in the men-to-women ratio across calendar years between city and mountain marathon running in Switzerland.

### Limitations and implications for future research

Findings from the ‘Jungfrau Marathon’ were compared to those of a ‘non-mountainous’ or ‘non-hilly’ city marathon. It would have been more appropriate to compare the ‘Jungfrau Marathon’ findings to those of a hilly marathon run mostly on paved surface. Then performance comparison would be more valid. Future studies might consider this aspect.

A further limitation is that the present findings are mainly relevant for Switzerland. Future studies might investigate other mountain marathons such as the ‘Rab Mountain Marathon’ (http://www.rabmountainmarathon.com) or the ‘Raccoon Mountain Marathon’ (http://runchattanooga.org/rmm/).

## Conclusions

In summary, performance decreased in age group athletes competing in ‘Jungfrau Marathon’ although participation increased. This finding for one of the most famous mountain marathons is different to findings for a large city marathon such as the ‘New York City Marathon’. These disparate findings might be explained by the different data analyses or as a typical phenomenon of races held in Switzerland. Future studies might investigate participation and performance trends in other mountain marathons or mountain ultra-marathons held in other countries such as Switzerland.
